# Efficacy and safety of antibody-drug conjugates in pretreated HER2-positive advanced breast cancer: a network meta-analysis of randomized controlled trials

**DOI:** 10.3389/fphar.2026.1921830

**Published:** 2026-09-28

**Authors:** Fen Liu, Yong Pan, Jinlan Li, Xiaojie Hu, Guisen Yin

**Affiliations:** 1 Department of Pharmacy, Hunan Cancer Hospital, The Affiliated Cancer Hospital of Xiangya School of Medicine, Central South University, Changsha, Hunan, China; 2 Department of Pharmacy, Yantai Hospital of Traditional Chinese Medicine, Yantai, Shandong, China

**Keywords:** antibody-drug conjugates, effectiveness, HER2 positive breast cancer, network meta-analysis, safety

## Abstract

**Background:**

Antibody-drug conjugates (ADCs) have become the standard treatment for HER2-positive(HER2+)advanced breast cancer following the failure of trastuzumab or taxane. Nevertheless, there are no direct head-to-head trials comparing different ADCs.

**Methods:**

We searched PubMed, Embase, the Cochrane Library and abstracts from major oncology conferences, and included phase III randomized controlled trials (RCTs) investigating HER2-targeting ADCs. We conducted a frequentist random-effects NMA, and utilized the P-score to rank the efficacy of included treatments. The outcome indicators include progression-free survival (PFS), overall survival (OS), and treatment-related adverse events (TRAEs).

**Results:**

Seven RCTs involving 3,160 patients and seven ADCs (T-DM1, ARX788, A166, SHR-A1811, RC48, DP303c and T-DXd) were included in our research. All ADCs showed a significant PFS benefit versus chemotherapy plus tyrosine kinase inhibitor (Chem + TKI). T-DXd showed the most favorable PFS (HR = 0.18, 95%CI: 0.13–0.25), followed by SHR-A1811 (HR = 0.22, 95%CI: 0.15–0.33) compared with Chem + TKI. SHR-A1811 showed a promising OS signal(HR = 0.31, 95% CI: 0.14–0.69), followed by T-DXd (HR = 0.41, 95% CI: 0.26–0.66) compared with Chem + TKI. For safety, T-DXd exhibited the highest odds for all-grade TRAEs relative to all included control treatments, with statistically significant differences identified when compared with T-DM1 and Chem + TKI., T-DM1 demonstrated better safety characteristics for grade ≥3 TRAEs, with a significantly lower odds of occurrence than SHR-A1811, ARX788, DP303c, and Chem + TKI.

**Conclusion:**

Included anti-HER2 ADCs significantly improve PFS over Chem + TKI, among which T-DXd and SHR-A1811 deliver notable survival benefits. Although T-DXd is associated with a higher odd of all-grade TRAEs and T-DM1 exhibits favourable grade ≥3 safety.

**Systematic Review Registration:**

https://www.crd.york.ac.uk/prospero/display_record.php?ID=CRD420251234345, identifier CRD420251234345.

## 1 Introduction

Over the past decade, the rapid advancement of antibody-drug conjugates (ADCs) has completely transformed the treatment landscape for advanced HER2+ breast cancer (Dai et al., 2023). Trastuzumab emtansine (T-DM1), as the first ADC approved for the treatment of HER2+ metastatic breast cancer, has become a second-line treatment option after the failure of trastuzumab therapy (Lambert and Chari, 2014). The DESTINY-Breast 01 study (Modi et al., 2020) demonstrated the remarkable efficacy of trastuzumab deruxtecan (T-DXd) in later-line settings. The DESTINY-Breast02 trial further confirmed T-DXd’s superiority over physician’s choice of therapy in patients with ≥1 prior anti-HER2 regimens, supporting its approval for later-line use (Fehm et al., 2024). The confirmatory phase III study within the T-DXd DESTINY clinical development program subsequently demonstrated that T-DXd significantly outperformed T-DM1 in both progression-free survival (PFS) and overall survival (OS) among patients with prior trastuzumab and taxane exposure, providing the evidentiary basis for its second-line global approval (Cortés et al., 2024). Meanwhile, novel domestic ADCs such as Disitamab vedotin (RC48), SHR-A1811, ARX788, and DP303c have also shown good therapeutic effects in phase III clinical trials (Wang et al., 2025; Song et al., 2025; Hu et al., 2025a; Hu et al., 2026).

However, at present, no adequate head-to-head randomized controlled trials (RCTs) have been carried out to enable direct comparative analysis across different ADCs. For breast cancer patients who have failed treatment with trastuzumab or taxane, there is still uncertainty in clinical decision-making regarding how to scientifically select ADCs (de Almeida et al., 2026). Meanwhile, there are significant differences in safety among different ADCs, and the adverse events linked to these therapies may directly compromise the health-related quality of life of the treated patients (Perachino et al., 2025). This study aims to conduct a systematic review and network meta-analysis (NMA) to clarify the efficacy and safety ranking of different ADCs for HER2+ advanced breast cancer patients after failure of trastuzumab or taxane-based treatment, thereby helping patients obtain a better treatment experience.

## 2 Materials and methods

### 2.1 Search strategy and screening criteria

Before conducting the NMA, we had already registered in PROSPERO in accordance with the PRISMA “Preferred Reporting Items for Systematic Reviews and Meta-Analyses” guidelines, with the registration number being CRD420251234345 (Hutton et al., 2015). In this study, we performed a comprehensive systematic literature search across three major academic databases (PubMed, Embase, and Cochrane Library) to obtain prospective clinical trials evaluating ADCs in patients with HER2+ advanced breast cancer. The search scope covered all records from the establishment of each database to 31 December 2025. Additionally, manual searches and screenings were conducted for conference abstracts published at the American Society of Clinical Oncology, European Society for Medical Oncology, and San Antonio Breast Cancer Symposium annual meetings from June 2020 to December 2025 to assess whether they met the inclusion criteria. Detailed search strategies for each database are provided in Supplementary Table S3. We verified all the included abstracts and only included English-language literature. The literature screening processwas shown in Figure 1 (PRISMA flowchart) (Moher et al., 2010).

**FIGURE 1:**
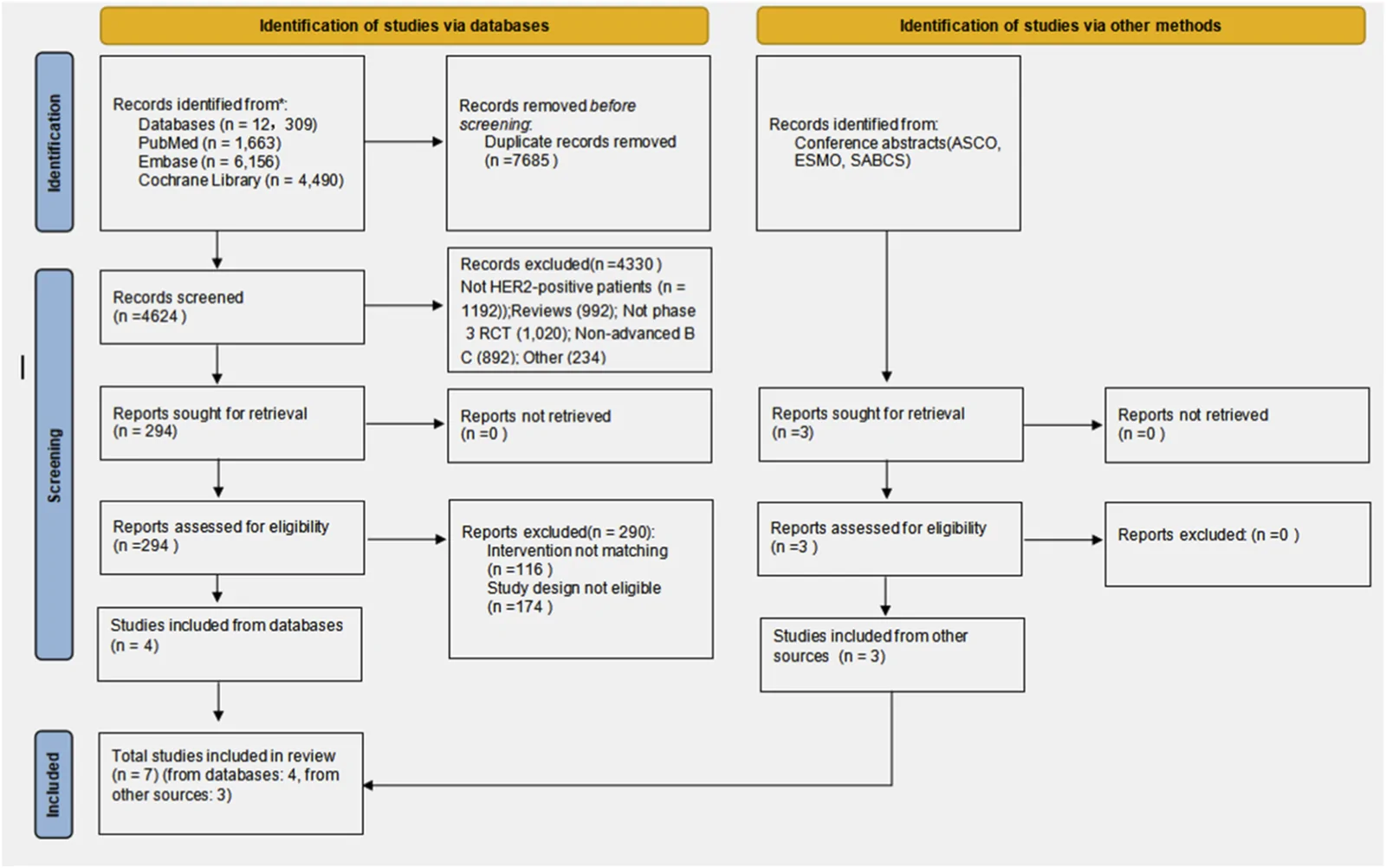
Flow chart of the detailed literature screening process. Abbreviations: BC, breast cancer; ASCO, American Society of Clinical Oncology; ESMO, European Society for Medical Oncology; SABCS, San Antonio Breast Cancer Symposium.

Inclusion criteria for clinical trials: (1) Phase III RCTs. (2) Patients diagnosed with HER2+ advanced breast cancer, who have been previously treated with trastuzumab or received systemic therapy built on taxane backbone.(3) At least one HER2-targeted ADC is included in the trial group or control group. (4) Detailed results and toxic effects, including PFS, and grade 3 or higher treatment-related adverse events (TRAEs), and OS when available, were reported in the study. For studies lacking OS data or unable to obtain such data, they can still be included in the analysis to evaluate the situation of recurrence free survival time and safety. Studies that do not meet the above criteria will be excluded.

### 2.2 Data extraction

Two researchers (F.L. and G.Y.) independently retrieved and screened the trials based on the inclusion and exclusion criteria. If multiple studies reported the same research, the latest data was taken as the standard. Any disputes were resolved by the third researcher (Y.P.) Key data, including the first author, publication year, study title, NCT number, sample size, intervention and control measures, number of previous anti-tumor treatment lines, primary efficacy endpoints, and TRAEs, were systematically extracted and recorded in Microsoft Excel. The extraction work was independently conducted by the two researchers (F.L. and G.Y.), and any differences were resolved by the third researcher (Y.P.) Additionally, if necessary, we would consider contacting the authors to obtain the data. If the data could not be obtained, the study would be excluded. Two independent reviewers (J.L. and X.H.) used the Cochrane Risk of Bias Assessment Tool Version 2.0 (RoB 2) to conduct risk-of-bias appraisal for randomized controlled trials (Sterne et al., 2019). Disagreements were resolved by the third reviewer (Y.P.)

### 2.3 Statistical analysis

We performed a network meta-analysis (NMA) within the frequentist statistical framework, adopting a random-effects model to estimate effect sizes (hazard ratio [HR] or odds ratio [OR]) and their corresponding 95% confidence intervals Chemotherapy plus tyrosine kinase inhibitor (Chem + TKI) was prespecified as the reference treatment for all comparisons. Transitivity assumptions were assessed by comparing the distribution of key potential effect modifiers across the included studies, including the number of previous lines of therapy, hormone receptor status, and control regimen. As shown in Table 1, the proportion of patients who had received two or more prior lines of therapy ranged from 11.30% to 59.70% across the included trials, and all the trials included patients with HER2+ advanced breast cancer who had received previous treatment with trastuzumab or a taxane. All the included trials reported data on subgroups of hormone-receptor status, and the adjudication criteria were consistent. We further conducted subgroup analyses based on this variable. Control regimens were generally homogeneous, mainly Chem + TKI or T-DM1. Based on the above assessment, the indirect comparison of this NMA basically satisfies the transitivity assumption.

**TABLE 1:** Baseline characteristics and outcomes of the included RCTs.

Study	Author, published year	Regimen (number of patients)		Median PFS (HR, 95% CI)	Median OS (HR, 95% CI)	Previous treatment line(≥2 line,%)	Follow-up time(months)
		Interventional group	Control group				
EMILIA	Diéras et al., 2017	T-DM1 (495)	Chem + TKI (496)	9.60 vs. 6.40 (0.65,0.55-0.77)	29.9 vs. 25.9 (0.75,0.64-0.88)	39.00	19.10 vs. 18.60
E-breast-02	Hu et al., 2025a	ARX788 (221)	Chem + TKI (220)	11.30 vs. 8.20 (0.64,0.49-0.82)	NR vs. NR (0.71,0.46-1.10)	33.50	11.10
KL166-III-06	Hu et al., 2025b	A166 (182)	T-DM1 (183)	11.10 vs. 4.40 (0.39,0.30-0.51)	NR vs. NR (0.62,0.38-1.03)	53.80	14.90
HORIZON-Breast01	Song et al., 2025	SHR-A1811 (142)	Chem + TKI (145)	30.60 vs. 8.30 (0.22,0.15-0.34)	NR vs. NR (0.31,0.14-0.69)	16.20	15.90 vs. 15.30
DESTINY-Breast03	Cortés et al., 2024	T-DXd (261)	T-DM1 (263)	NR vs. 6.80 (0.28,0.22-0.37)	NR vs. NR (0.55,0.36–0.86)	49.40	16.20 vs. 15.30
RC48-C006	Wang et al., 2025	RC48 (53)	Chem + TKI (51)	NR vs. NR 0.56(0.350-0.90)^a^	NR vs. NR 0.56(0.35-0.90)^a^	11.30	15.38 vs. 19.35
NCT06313086	Hu et al., 2026	DP303c (226)	T-DM1 (222)	8.80 vs. 5.80 (0.56,0.42,0.75)	NR	59.70	7.40

Chem + TKI, chemotherapy combined with tyrosine kinase inhibitors; CI, confidence interval; HR, hazard ratio; NR, not available; OS, overall survival; PFS, progression-free survival; T-DM1, trastuzumab emtansine; A166, Trastuzumab botidotin; T-DXd, trastuzumab deruxtecan; RC48, disitamab Vedotin

^a^ As this study exclusively enrolled HER2-positive advanced breast cancer with liver metastasis, the corresponding data were incorporated solely into the visceral metastasis subgroup analysis and excluded from the overall population analysis of the study.

For survival outcomes, an HR < 1 indicated that the experimental group performed better than the reference treatment; for safety outcomes, an OR < 1 suggested a lower odd of adverse events in the experimental group (i.e., greater safety) (Guo et al., 2024). In the evidence network of this study, each direct comparison is supported by only one independent single study, and there is no closed loop among the various treatment measures. Therefore, it is impossible to effectively estimate the heterogeneity parameters (I^2^,τ^2^) using conventional methods, nor can the node-splitting method be used to formally evaluate the consistency between direct and indirect evidence (Schettini et al., 2025). To partially overcome this methodological limitation, we pre-specified the use of a random effects model. Specifically, because data from only individual studies contributed to each direct comparison, we estimated the between-study variance (τ^2^) on the basis of the entire evidence network using the DerSimonian-Laird method, which is implemented by default in R packages. To further test the robustness of the results, we conducted a “leave-one-out” sensitivity analysis to assess the impact of eliminating each individual trial on the pooled effect size and treatment ranking. The results are shown in Supplementary Figure S3. Additionally, we also conducted subgroup analyses for the HR-/HER2+ population and the visceral metastasis population to verify the reliability of the main analysis results. All analyses were performed using the R software package ‘netmeta`.

The direct comparison relationships among various intervention measures are presented through a network diagram, where the size of the nodes represents the total sample size of each intervention group, and the thickness of the lines represents the number of studies for the direct comparisons (Schwingshackl et al., 2018). We utilized the P-score, a frequentist ranking indicator for NMA, to sort all included intervention measures, and a greater P-score value denoted both more favorable therapeutic efficacy and a lower risk of treatment-related adverse events (Rosenberger et al., 2021). The HR/OR and 95%CI matrix for all pairwise comparisons of treatments was generated through the league table (Du et al., 2021).

## 3 Results

### 3.1 Study characteristics and quality evaluation

The process of literature search and screening, as well as the characteristics of the included studies, were separately illustrated in Figure 1 and summarized in Table 1. We included a total of seven phase III randomized controlled trials, involving 3,160 patients, including 8 treatment groups and 7 targeted HER2 ADCs, namely T-DM1 (Diéras et al., 2017), ARX788 (Hu et al., 2025a), A166 (Hu et al., 2025b), SHR-A1811 (Song et al., 2025), RC48 (Wang et al., 2025), DP303c (Hu et al., 2026), and T-DXd (Gogia and Ganguly, 2022). All the studies were randomized controlled phase III clinical trials for HER2+ advanced breast cancer that had failed previous treatment with trastuzumab or taxane. Among them, the RC48-C006 (Wang et al., 2025) study only included patients with liver metastasis from HER2+ advanced breast cancer, so this study only included subgroup analysis of visceral metastasis. The PFS NMA for all populations included 6 other studies (Song et al., 2025; Hu et al., 2025a; Hu et al., 2026; Diéras et al., 2017; Hu et al., 2025b; Gogia and Ganguly, 2022) except for the RC48-C006 study. Due to the missing OS data in the DP303c study, the OS NMA for the entire population included 5 other studies (Song et al., 2025; Hu et al., 2025a; Diéras et al., 2017; Hu et al., 2025b; Gogia and Ganguly, 2022) except for the RC48-C006 (Wang et al., 2025) and DP303c studies (Hu et al., 2026).

We applied the Cochrane RoB 2 tool to assess the risk of bias of the included RCT, and the results are presented in Supplementary Figure S1. Four studies (A166, RC48, SHR-A1811, DP303c) had incomplete reporting of randomisation details, resulting in some concerns for the randomisation process domain, the remaining three trials were rated low risk in this domain.All seven trials had an open-label design. No deviations from the intended interventions associated with knowledge of treatment allocation were identified, and appropriate intention-to-treat (ITT) analyses were performed. Therefore, the domain of deviations from intended interventions was judged as low risk across all studies. The primary endpoint was adjudicated by a blinded independent central review (BICR) with reviewers masked to treatment assignment. Accordingly, the domain of measurement of the outcome was rated low risk for all trials.For the domain of missing outcome data: the three fully published trials were rated low risk owing to the availability of full reporting of censoring and missing data. For the four trials available only as conference abstracts (A166, RC48, SHR-A1811, DP303c), exact counts and reasons for missing outcome data were not reported, precluding independent verification of the completeness of outcome data; these four studies were thus assigned some concerns. For the domain of selection of the reported result, studies without publicly available statistical analysis plans (SAPs, mostly conference abstracts) raised some concerns, whereas trials with accessible full protocols/SAPs were rated low risk. Overall, the included studies were judged to have either low risk or some concerns, and no trial was rated high risk in any domain. The concerns mainly stemmed from incomplete descriptions of randomisation, unavailable public SAPs, and limited reporting of missing outcome data for several conference-abstract trials.

### 3.2 Network meta-analyses for efficacy

The primary research objectives were the PFS analysis and OS analysis for the overall populations. Regarding the PFS analysis of the overall population, a total of seven intervention measures (Chem + TKI, A166, ARX788, DP303c, SHR-A1811, T-DM1, T-DXd) were included, covering six direct comparisons. The network diagram (Figure 2A) showed multiple direct comparisons among the intervention measures. The table of league (Table 2) presented the pairwise comparison results among all treatment groups. Compared with the reference treatment, all treatment groups showed a PFS advantage (HR < 1), and this difference reached statistical significance. Among them, T-DXd had the most significant PFS advantage (HR = 0.18, 95% CI: 0.13-0.25), followed by SHR-A1811 (HR = 0.22, 95% CI: 0.15-0.33). As shown in Figure 3, the P-score indicated the ranking of PFS advantages among the treatment groups: T-DXd (0.95) > SHR-A1811 (0.82) > A166 (0.72) > DP303c (0.51) > ARX788 (0.26) > T-DM1 (0.24) > Chem + TKI (0.00).

**FIGURE 2:**
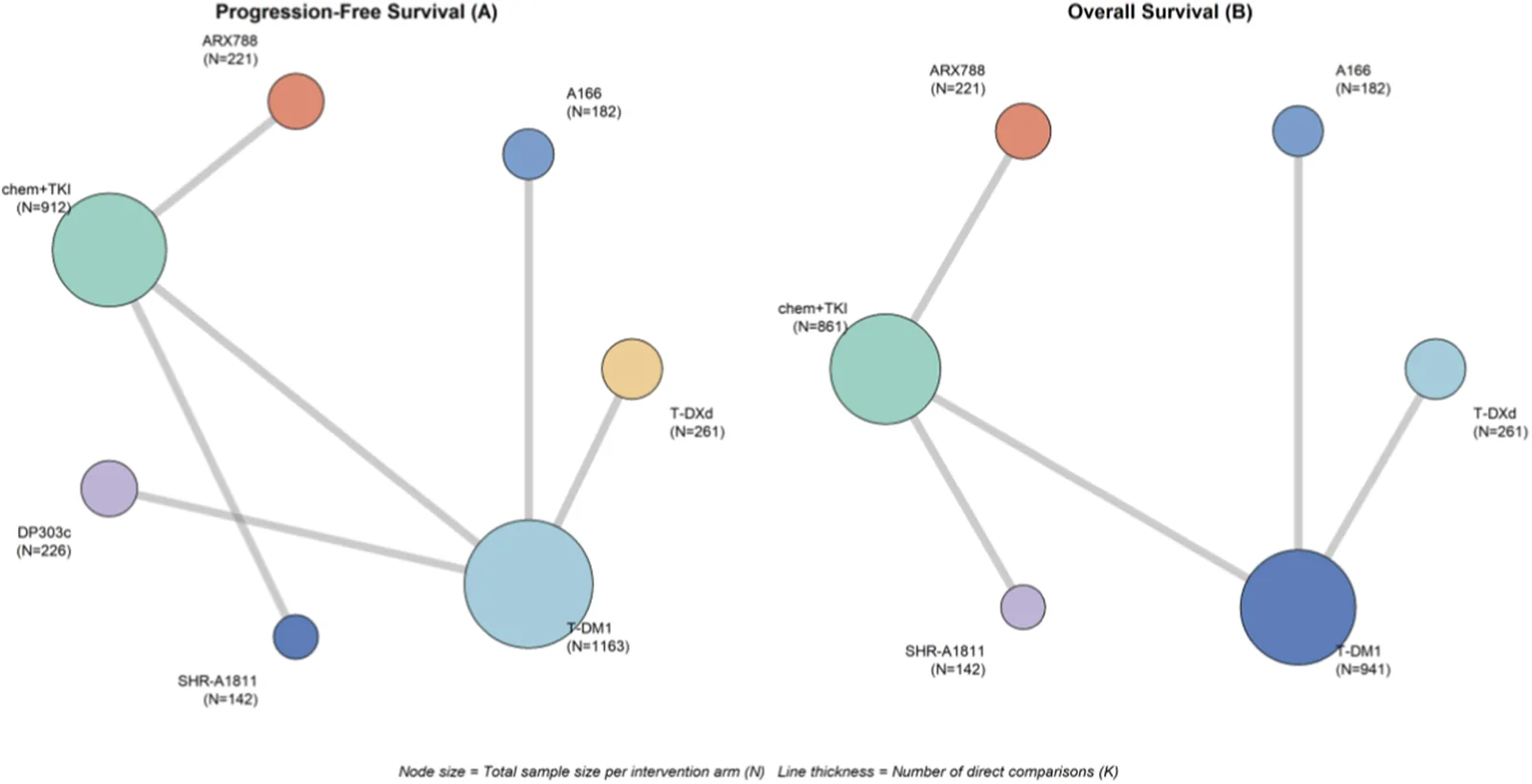
Network plot of PFS **(A)** and OS **(B)** for ADCs in patients with HER2-positive advanced breast cancer who failure of trastuzumab or taxane. Every node represents one intervention, and the lines denote direct comparisons. No closed loops exist in the networks, indicating that all comparisons are either direct or indirect without a combination of both for any treatment pair. Abbreviations: PFS, progression-free survival; OS, overall survival; ADCs, antibody-drug conjugates; T-DM1, trastuzumab emtansine; A166, Trastuzumab botidotin; T-DXd, trastuzumab deruxtecan; RC48, disitamab Vedotin; Chem + TKI, chemotherapy combined with tyrosine kinase inhibitors.

**TABLE 2:** Pairwise comparisons among different ADCs for PFS (upper right) and OS (lower left) in the overall population.

**T-DXd**	0.83 (0.50-1.38)	0.72 (0.50-1.04)	**0.50 (0.34-0.74)**	**0.28 (0.19-0.43)**	**0.28 (0.22-0.36)**	**0.18 (0.13-0.25)**
1.33 (0.53-3.35)	**SHR-A1811**	0.87 (0.52-1.45)	0.60 (0.36-1.03)	**0.34 (0.21–0.56)**	**0.34 (0.22–0.53)**	**0.22 (0.15–0.33)**
0.89 (0.46-1.72)	0.67 (0.26-1.73)	**A166**	0.70 (0.47-1.03)	**0.40 (0.26–0.59)**	**0.39 (0.30–0.51)**	**0.25 (0.19–0.35)**
NA	NA	NA	**DP303c**	**0.57 (0.37–0.87)**	**0.56 (0.42–0.75)**	**0.36 (0.26–0.51)**
0.58 (0.31-1.10)	0.44 (0.18-1.08)	0.65 (0.33-1.29)	NA	**ARX788**	0.98 (0.72-1.34)	**0.64 (0.49–0.83)**
**0.55 (0.36–0.85)**	0.41 (0.18-0.93)	0.62 (0.38-1.02)	NA	0.95 (0.60-1.51)	**T-DM1**	**0.65 (0.55–0.77)**
**0.41 (0.26–0.66)**	**0.31 (0.14–0.69)**	**0.47 (0.28–0.78)**	NA	0.71 (0.46-1.10)	**0.75 (0.64–0.88)**	**Chem + TKI**

Data are represented as hazard ratios and corresponding 95% confidence intervals (CIs). The HR, value at the left lower is column-to-row ratio, and the HR, at the upper right is row-to-column ratio. Data with statistical significance are marked in bold. NA, not available; T-DM1, trastuzumab emtansine; A166, Trastuzumab botidotin; T-DXd, trastuzumab deruxtecan; RC48, disitamab Vedotin; Chem + TKI, chemotherapy combined with tyrosine kinase inhibitors.

**FIGURE 3:**
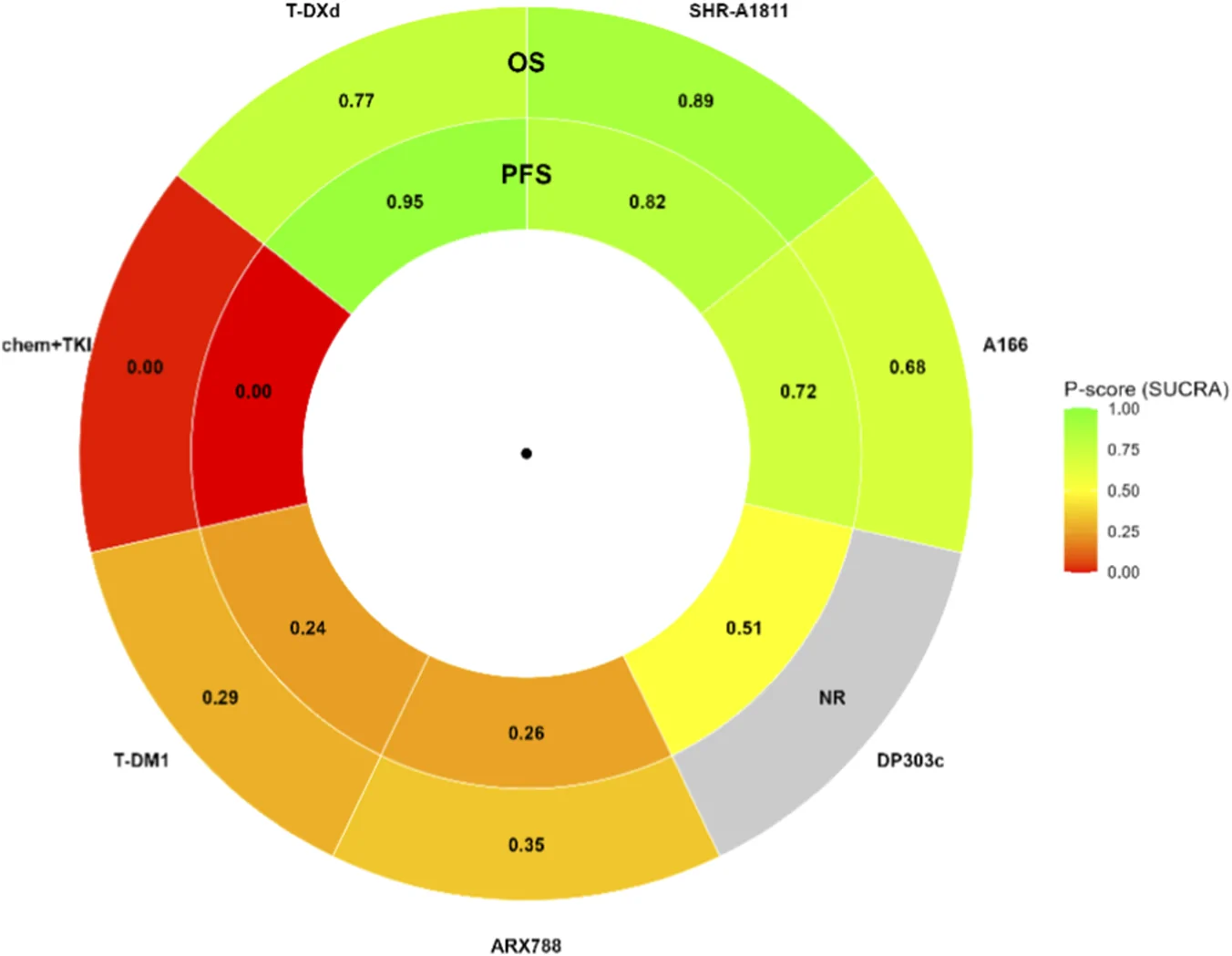
Heatmap of P-scores for PFS and OS in the overall population. The P-score is indicated by the shade of color (0-1). The darker the color, the higher the P-score and the better the therapeutic effect. The values within each cell represent the corresponding P-score. Abbreviations: PFS, Progression-Free Survival; OS, Overall Survival; NA, not available; T-DM1, trastuzumab emtansine; A166, Trastuzumab botidotin; T-DXd, trastuzumab deruxtecan; RC48, disitamab Vedotin; Chem + TKI, chemotherapy combined with tyrosine kinase inhibitors.

Regarding the OS analysis for the overall population, a total of six intervention measures (Chem + TKI, A166, ARX788, SHR-A1811, T-DM1, T-DXd) were included, covering 5 direct comparisons (Figure 2B). Table 2 showed that all treatment groups except ARX788 demonstrated an OS advantage (HR < 1) with statistical significance difference compared to the reference treatment. Among them, SHR-A1811 showed the most prominent OS benefit (HR = 0.31, 95% CI: 0.14-0.69), followed by T-DXd (HR = 0.41, 95% CI: 0.26-0.66). The P-score ranking results (Figure 3) were consistent with the league table data. SHR-A1811 was the most likely to have the best OS advantage (0.89), followed by T-DXd (0.77) among the included ADCs. It should be noted that the OS data from multiple clinical trials (such as HORIZON-Breast01, KL166-III-06) were not yet mature; therefore, the observed OS ranking is a hypothetical exploration result rather than a definitive conclusion.

In addition, we also analyzed the PFS of the subgroups of HR-/HER2+ and visceral metastasis. For the HR-/HER2+ population, a total of five intervention measures (Chem + TKI, ARX788, SHR-A1811, T-DM1, T-DXd) were included in four direct comparisons. The league table (Supplementary Table S1) showed the pairwise comparison results among all treatment groups. Compared with the reference treatment, all treatment groups showed a PFS advantage (HR < 1) with significance statistical difference. Among them, the PFS advantage of T-DXd was the most significant (HR = 0.17, 95% CI: 0.11-0.27), followed by SHR-A1811 (HR = 0.23, 95% CI: 0.13-0.42), consistent with the results from the entire population. The P-score results (Supplementary Figure S2) were consistent with the league table results. Regarding the visceral metastasis population, a total of six intervention measures (Chem + TKI, ARX788, SHR-A1811, T-DM1, T-DXd, RC48) were included in five direct comparisons. The league table (Supplementary Table S1) and the P-score results (Supplementary Figure S2) also indicated that the PFS advantage of T-DXd was the most prominent, followed by SHR-A1811 among the included ADCs.

Overall, whether in the HR-/HER2+ and visceral metastasis subgroup population, or in the overall population, T-DXd demonstrated the most prominent PFS benefit relative to the reference treatment among the included ADCs, followed by SHR-A1811. Among all the ADCs included in the comparison, SHR-A1811 demonstrated a more significant OS advantage relative to the reference treatment, followed by T-DXd.

### 3.3 Network meta-analyses for safety

For the safety analysis, a total of eight intervention measures (Chem + TKI, A166, ARX788, DP303c, SHR-A1811, T-DM1, T-DXd, RC48) were included for seven direct comparisons to analyze all-grade TRAEs and grade ≥3 TRAEs. As shown in Table 3, T-DXd exhibited the highest odds for all-grade TRAEs relative to all included control treatments, with statistically significant differences identified when compared with T-DM1 and Chem + TKI. Specifically,the incidence of all-grade TRAEs with T-DXd was 7.86 times higher than that of T-DM1(OR = 7.86, 95%CI: 3.03-20.40), and 4.23 times than that of Chem + TKI (OR = 4.23, 95%CI: 1.26-14.22), with the difference reaching statistical significance; For grade ≥3 TRAEs, T-DM1 demonstrated better safety characteristics, with a significantly lower odds of occurrence than SHR-A1811 (OR = 0.44, 95%CI: 0.25-0.76), ARX788 (OR = 0.58, 95%CI: 0.37-0.92), DP303c (OR = 0.44, 95%CI: 0.30 -0.65), and Chem + TKI (OR = 0.61, 95%CI: 0.48-0.79). It is worth noting that the odds of TRAEs ≥3 in SHR-A1811group exhibited a trend of being higher than that of in all control treatments, and the difference compared with T-DM1 reached statistical significance(OR = 2.29, 95%CI: 1.32-3.98).

**TABLE 3:** Pairwise comparisons among different ADCs for all-grade (upper right triangle) and grade ≥3 (lower left triangle) treatment-related adverse events in the overall population.

**SHR-A1811**	0.60 (0.02-16.23)	0.49 (0.01-19.26)	0.30 (0.01-17.83)	0.29 (0.00-17.41)	0.32 (0.01-8.02)	0.10 (0.00-9.64)	0.08 (0.00-2.36)
**2.29 (1.32–3.98)**	**T-DM1**	0.81 (0.12-5.67)	0.50 (0.04-5.56)	0.49 (0.04-5.43)	0.54 (0.26-1.14)	0.17 (0.01-4.64)	**0.13 (0.05–0.33)**
1.33 (0.71-2.48)	**0.58 (0.37–0.92)**	**ARX788**	0.62 (0.03-13.71)	0.61 (0.03-13.39)	0.67 (0.11-4.03)	0.21 (0.01-8.41)	0.16 (0.02-1.38)
1.76 (0.87-3.56)	0.77 (0.50-1.19)	1.32 (0.70-2.49)	**A166**	0.98 (0.03-29.48)	1.08 (0.09-13.42)	0.34 (0.01-20.31)	0.25 (0.02-3.40)
1.01 (0.52-1.98)	**0.44 (0.30–0.65)**	0.76 (0.42-1.38)	0.57 (0.32-1.02)	**DP303c**	1.10 (0.09-13.70)	0.35 (0.01-20.76)	0.26 (0.02-3.47)
1.41 (0.86-2.30)	**0.61 (0.48–0.79)**	1.06 (0.72-1.55)	0.80 (0.48-1.32)	1.39 (0.88-2.20)	**Chem + TKI**	0.31 (0.01-7.90)	**0.24 (0.07–0.79)**
1.27 (0.50-3.22)	0.55 (0.24-1.27)	0.95 (0.40-2.30)	0.72 (0.28-1.84)	1.26 (0.50-3.13)	0.90 (0.41-1.99)	**RC48**	0.75 (0.02-23.50)
1.82 (0.95-3.49)	1.26 (0.89-1.78)	1.37 (0.77-2.43)	1.03 (0.59-1.80)	**1.80 (1.07–3.00)**	1.03 (0.59-1.80)	1.43 (0.58-3.52)	**T-DXd**

Data are represented as odds ratios and corresponding 95% confidence intervals (CIs). The OR, value at the left lower is column-to-row ratio, and the OR, at the upper right is row-to-column ratio. Data with statistical significance are marked in bold. NA, not available; T-DM1, trastuzumab emtansine; A166, Trastuzumab botidotin; T-DXd, trastuzumab deruxtecan; RC48, disitamab Vedotin; Chem + TKI, chemotherapy combined with tyrosine kinase inhibitors.

Additionally, we conducted a comparative analysis of adverse events leading to treatment discontinuation and dose reduction. As shown in Table 4, compared with T-DXd, SHR-A1811, ARX788, RC48 and Chem + TKI, the odds ratio for treatment discontinuation was significantly lower in the A166 group; However, the comparison with T-DM1 and DP303c did not reach statistical significance. In contrast, ARX788 was associated with a significantly higher odds of treatment discontinuation compared to Chem + TKI, T-DM1, T-DXd, DP303c, and A166, and the difference reached statistically significant; Furthermore, T-DM1 and SHR-A1811 exhibited lower odds of dose reduction, performing better than ARX788, RC48, A166, Chem + TKI, and DP303c, with statistically significant differences.

**TABLE 4:** Pairwise comparisons among different ADCs for adverse events leading to treatment discontinuation (lower left) and dose reduction (upper right).

**T-DM1**	NA	0.90 (0.50-1.63)	**0.27 (0.16-0.45)**	**0.29 (0.12-0.67)**	**0.21 (0.13-0.32)**	**0.14 (0.10-0.19)**	**0.13 (0.08-0.20)**
**0.51 (0.28–0.91)**	**T-DXd**	NA	NA	NA	NA	NA	NA
**0.15 (0.03–0.65)**	0.29 (0.06-1.42)	**SHR-A1811**	**0.30 (0.16–0.59)**	**0.32 (0.12–0.83)**	**0.23 (0.11–0.48)**	**0.16 (0.09–0.26)**	**0.14 (0.07–0.30)**
**0.13 (0.04–0.40)**	**0.26 (0.07–0.91)**	0.90 (0.15-5.43)	**ARX788**	1.05 (0.43-2.60)	0.76 (0.39-1.49)	**0.51 (0.34–0.78)**	**0.46 (0.23–0.92)**
0.06 (0.00-1.31)	0.13 (0.01-2.73)	0.45 (0.02-12.33)	0.50 (0.02-11.86)	**RC48**	0.72 (0.28-1.89)	0.49 (0.22-1.09)	0.44 (0.17-1.16)
3.07 (0.72-13.03)	**6.03 (1.27–28.66)**	**21.10 (2.63–169.12)**	**23.54 (3.76–147.47)**	**47.41 (1.68–1,338.55)**	**A166**	0.67 (0.40-1.15)	0.61 (0.32-1.15)
**0.46 (0.32–0.67)**	0.91 (0.46-1.81)	3.18 (0.74-13.55)	**3.55 (1.22–10.30)**	7.14 (0.36-141.74)	**0.15 (0.03–0.67)**	**Chem + TKI**	0.90 (0.52-1.55)
1.43 (0.28-7.33)	2.81 (0.50-15.94)	**9.85 (1.07–90.27)**	**10.99 (1.51–80.04)**	22.13 (0.72-680.33)	0.47 (0.05-4.14)	3.10 (0.58-16.54)	**DP303c**

Data are represented as odds ratios and corresponding 95% confidence intervals (CIs). The OR, value at the lower left is column-to-row ratio, and the OR, at the upper right is row-to-column ratio. Data with statistical significance are marked in bold. NA, not available; T-DM1, trastuzumab emtansine; A166, Trastuzumab botidotin; T-DXd, trastuzumab deruxtecan; RC48, disitamab Vedotin; Chem + TKI, chemotherapy combined with tyrosine kinase inhibitors.

A descriptive summary of TRAEs found distinct incidence patterns of individual adverse events across HER2-targeted therapeutic regimens (Supplementary Table S2). For hematologic adverse events, SHR-A1811 had the highest incidence of neutropenia, leukopenia, and anemia among all groups, but not the highest for thrombocytopenia. T-DM1 ranked first in any-grade thrombocytopenia incidence (41.15%) in this study. For gastrointestinal adverse events, T-DXd was linked to higher nausea and vomiting incidence, while the CT + TKI regimen had a significantly higher diarrhea incidence than ADCs. Elevated hepatic enzyme levels occurred across all groups, with ARX788 having the highest incidence of alanine aminotransferase (ALT) and aspartate aminotransferase (AST) elevations. ARX788 was associated with characteristic ocular adverse events, and notably its interstitial lung disease incidence (32.7%) was markedly higher than other regimens. Hand-foot syndrome was more frequent in the Chemo + TKI group. Comprehensive safety profiles of A166 and DP303 have not been reported publicly, and available safety data for the two agents remains limited. This section only provides descriptive summaries of individual adverse event incidence extracted directly from included original studies. No network meta-analysis was performed for indirect comparison of safety outcomes across interventions, so no statistically robust inferences can be made on the relative adverse event risk differences of the evaluated agents.

## 4 Discussion

This study, through a NMA, systematically compared the efficacy and safety of seven ADCs in HER2+ advanced breast cancer patients who had failed previous trastuzumab or taxane-based treatment. The results demonstrated that, among the overall population, all ADCs yielded a statistically significant PFS benefit compared with the Chem + TKI group. Among them, T-DXd conferred the most prominent advantage, with HR of 0.18 (95% CI: 0.13-0.25), followed by SHR-A1811 (HR = 0.22, 95% CI: 0.15-0.33). In terms of OS, SHR-A1811 showed a favorable OS benefit trend(HR = 0.31, 95% CI: 0.14–0.69) compared with the Chem + TKI group, followed by T-DXd (HR = 0.41, 95% CI: 0.26–0.66). Due to the immature OS data from several key trials (HORIZON-Breast01, KL166-III-06), the observed OS ranking should be interpreted with caution, and this result should only be regarded as a hypothesis until the updated survival analysis results are released. Subgroup results demonstrated that in patients with HR-/HER2+ or visceral metastasis, similar to the entire population, T-DXd showed the most significant PFS advantage compared to the reference treatment, followed by SHR-A1811. Descriptive safety data revealed divergent patterns of individual adverse events across different HER2-targeted regimens. Given that separate indirect comparisons were not conducted for each individual adverse event, coupled with substantial between-trial heterogeneity across the included studies, interpretation of the observed toxicity profiles should be approached with caution. Clinical decision-making should balance treatment benefits and risks in conjunction with efficacy outcomes, and clinicians should strengthen monitoring for regimen-specific adverse events.

Zian Kang et al. (Kang et al., 2024) conducted a conventional meta-analysis and found that, ADCs significantly outperformed the Chem + TKI or other comparator regimens in patients with HER2+ advanced breast cancer. However, this study only included T-DM1, SYD985, and T-DXd, and used a traditional Meta-analysis method, thus unable to compare the relative efficacy rankings among different ADCs. Additionally, this study did not include the latest 2025 clinical trials of SHR-A1811, A166, DP303c, and ARX788. Our study is the first to incorporate the III-stage trial data of seven ADCs, including four domestic ADCs, through a NMA to achieve an indirect comparison between T-DXd and domestic ADCs, and comprehensively evaluated the adverse reactions of each drug. Our research not only systematically evaluated the effectiveness of ADCs in the later-line treatment of HER2+ advanced breast cancer, but also clarified the efficacy gradient among different ADCs and provided a basis for drug selection based on toxicity characteristics.

The DESTINY-Breast03 study confirmed that T-DXd showed significant PFS benefits (HR = 0.28) and OS benefits (HR = 0.64) compared to T-DM1 in patients with disease progression following prior trastuzumab or taxane-based treatment. This study expanded the PFS advantage of T-DXd to comparisons with various ADCs, further consolidating its position as the preferred choice for late-line treatment. In terms of OS, SHR-A1811 demonstrated a favorable trend relative to comparator arms—potentially attributable to its higher drug-to-antibody ratio and optimized payload release kinetics (Yao et al., 2026; Zhang et al., 2025). However, this improvement did not reach statistical significance when compared with all other treatments except the reference treatment group. Since the results of the III-phase clinical trial of this drug are not yet fully mature, updated data are still needed to support the existing research results (Xie et al., 2025). The III-phase study of DP303c showed that its PFS was superior to T-DM1 (HR = 0.56), and our analysis found that its PFS ranking was in the middle with a moderate P-score of 0.51, making it one of the alternative choices for T-DM1. RC48 showed a PFS advantage in the HER2+ patients with liver metastasis (HR = 0.56), and its PFS ranking in the visceral metastasis subgroup with a P-score of 0.46 was consistent with this, supporting its application in patients with breast cancer with liver metastasis.

This study found that T-DXd demonstrated the most significant efficacy in terms of PFS, while SHR-A1811 exhibited a promising trend in OS benefits, which did not reach statistical significance except in the reference treatment group. This observation presents a “PFS-OS dissociation” phenomenon. This phenomenon is not uncommon in clinical research and may be caused by the following reasons: Firstly, the sensitivity of PFS and OS to treatment effects is different (Cerou et al., 2024). PFS reflects the initial inhibitory effect of treatment on tumor growth, while OS mainly evaluates the long-term treatment effect of patients. It is susceptible to multiple factors, including but not limited to subsequent treatment regimens, cross-treatment, and the patient’s disease progression (Larkin et al., 2023; U.S. Food and Drug Administration, 2025). In this study, T-DXd showed a relatively significant effect in PFS (HR = 0.18). If patients adopt other treatment regimens in subsequent lines of treatment, this may weaken the advantage of T-DXd in terms of OS. Secondly, the current OS data for SHR-A1811 are not yet mature, and it is not possible to clearly state that SHR-A1811’s OS is definitely superior to T-DXd’s. Future updated data are needed to support the above results. Thirdly, different ADCs loading mechanisms may also affect the ranking of PFS and OS (Fu et al., 2022). Since the subgroup analysis in this study was limited to PFS and the corresponding OS subgroup analysis data were lacking, whether this separation phenomenon is also applicable to the other subgroups cannot be definitively concluded.

### 4.1 Limitations

Firstly, all comparisons are indirect comparisons, and more head-to-head trials between different ADCs are needed to confirm our findings. Secondly, the heterogeneity of previous treatment regimens among different studies may also affect the observed results (Boone et al., 2023). Thirdly, since the OS data of some clinical trials (such as HORIZON-Breast01, KL166-III-06) are not yet mature, future updates of clinical trial results are needed to verify the observed OS ranking results. Fourthly, the safety comparison in this study did not correct for the differences in baseline characteristics of patients among different trials, which may affect the incidence of ADR in patients. Future studies that adopt unified safety reporting and correction analysis are needed (Gao et al., 2026). Fifthly, subgroup analysis is limited by the small sample size, and there may not be sufficient statistical data to detect moderate differences. Therefore, this limitation may introduce potential biases and affect the reliability of subgroup results (Cuijpers et al., 2021; Bunn et al., 2020). Sixthly, the clinical trials included in this study were mainly evaluated in the Chinese population. Although the efficacy results are encouraging, the generalizability of these findings in Western or other racial populations still requires more regional trials to verify (Paget et al., 2023; Liu et al., 2022). Finally, we cannot rule out publication bias, although including all available III-phase trials can to some extent alleviate this concern. Additionally, a large number of paired comparisons were made in safety outcomes and subgroup analyses, and no formal correction for multiplicity was performed; therefore, some statistically significant differences may be coincidental, and the interpretation of these results should be conservative.

### 4.2 Clinical implications

When interpreting the results of this NMA to guide the selection of ADCs, both tumor diagnosis and molecular heterogeneity should be emphasized, and the impacts of individual patient-related risk factors on efficacy and toxicity should also be fully considered. Atypical HER2 status, as well as intra-tumoral and inter-lesional heterogeneity of antigen expression, can contribute to biopsy sampling bias and diagnostic misclassification, which leads to a subset of patients failing to derive sufficient clinical benefit from ADC therapy (Wilcock et al., 2025). Drug-resistant phenotypes mediated by extracellular vesicles (Jahangiri, 2025) and potential biomarkers such as PTPN3 (Thankachan et al., 2025) further indicate that intrinsic molecular features of tumors, beyond target-antigen expression, also modulate therapeutic responses to ADCs. On the other hand, treatment-related toxicities are constrained by individual baseline patient characteristics, and drug selection should not rely merely on safety rankings. A pharmacovigilance study investigating risk factors for zolbetuximab-associated nausea and vomiting (Nagase and Shinozaki, 2025) serves as an example: even among targeted therapeutic agents, the risk of adverse events varies substantially across individuals. In conclusion, the efficacy and safety rankings derived from this study represent only population-level statistical probabilities. In clinical practice, comprehensive assessment integrating tumor molecular phenotypes, diagnostic limitations and individual patient risk factors is required to facilitate individualized therapeutic strategies for ADCs.

## 5 Conclusions

This NMA indicated that all included anti-HER2 ADCs conferred statistically significant PFS benefits compared with Chemo + TKI regimen. Among the ADCs evaluated in the present study, T-DXd and SHR-A1811 demonstrated particularly prominent PFS advantages. However, OS data from the currently included studies remains immature; as a consequence, the ranking results generated from the currently available OS data are preliminary and hypothesis-generating. Interpretation of these results requires cautious consideration, pending confirmation from updated and mature OS datasets. T-DXd was associated with a higher risk of all-grade TRAEs, whereas T-DM1 demonstrated a more favourable safety profile with respect to grade ≥3 TRAEs. These findings may inform clinical decision-making for HER2-positive breast cancer treatment. Nevertheless, tumour molecular heterogeneity and individual patient-related risk factors should also be taken into account when selecting ADCs for clinical application.

## Data Availability

The original contributions presented in the study are included in the article/Supplementary Material, further inquiries can be directed to the corresponding author.
