# Supplementary material for: Efficacy and safety of antibody-drug conjugates in pretreated HER2-positive advanced breast cancer: a network meta-analysis of randomized controlled trials
Source: Front Pharmacol. 2026 Sep 28;17:1921830. doi: 10.3389/fphar.2026.1921830 (PMC13655522; doi:10.3389/fphar.2026.1921830)
Supplement: Supplementary file 1 [file Supplementaryfile1.docx]

**Supplement**

A

B

Figure S1. Risk of bias assessment for RCTs according to the RoB 2 tool. (A) Risk of bias graph;

(B) Riskofbias summary.

Figure S2. Heatmap of P-scores for PFS in the HR-/HER2+ and visceral metastases subgroups.

The P-score is indicated by the shade of color (0-1). The darker the color, the higher the P-score and the better the therapeutic effect. The values within each cell represent the corresponding P-score. T-DM1, trastuzumab emtansine; T-DXd, trastuzumab deruxtecan; RC48, disitamab Vedotin; Chem + TKI, chemotherapy combined with tyrosine kinase inhibitors.

Figure S3.Sensitivity analysis: PFS and OS after excluding studies one by one. Each row represents the hazard ratio (HR) and 95% confidence interval (CI) corresponding to each ADCs after excluding one independent clinical trial. The dashed red line is the pooled hazard ratio from the original NMA analysis (all trials included). The solid black line represents the invalid reference HR=1. HR < 1 suggests that this ADC drug has better efficacy. The results of the PFS analysis remained robust under all exclusion scenarios. For OS outcomes, after excluding the KL166-III-06 study, the confidence interval of ARX788 crossed the null value (HR = 0.71, 95%CI 0.46-1.10), suggesting that the OS benefit of this drug needs to be interpreted with caution.

Table S1. Pairwise comparisons among different ADCs for PFS in patients with HR-/HER2+ disease (upper right triangle) and visceral metastases (lower left triangle).

| **T-DXd** | 0.73 (0.34 - 1.56) | **0.30 (0.20 - 0.44)** | NA | **0.27(0.15 - 0.45)** | **0.17 (0.11 - 0.27)** |
| --- | --- | --- | --- | --- | --- |
| 0.64 (0.40 - 1.0) | **SHR-A1811** | **0.41 (0.22 - 0.78)** | NA | **0.36 (0.18 - 0.74)** | **0.23 (0.13 - 0.42)** |
| **0.28 (0.22 - 0.36)** | **0.44 (0.29 - 0.65)** | **T-DM1** | NA | 0.89 (0.56 - 1.41) | **0.56 (0.44 - 0.72)** |
| **0.28 (0.16 - 0.47)** | **0.43 (0.25 - 0.74)** | 0.98 (0.61 - 1.57) | **RC48** | NA | NA |
| **0.24 (0.16 - 0.37)** | **0.38 (0.24 - 0.59)** | 0.86 (0.61 - 1.21) | 0.88 (0.52 - 1.46) | **ARX788** | **0.63 (0.43 - 0.93)** |
| **0.15 (0.11 - 0.21)** | **0.24 (0.17 - 0.34)** | **0.55 (0.45 - 0.67)** | **0.56(0.36 - 0.86)** | **0.64 (0.48 - 0.85)** | **Chem+TKI** |

Data are represented as hazard ratios and corresponding 95% confidence intervals (CIs). The HR value at the **left lower is column-to-row ratio**, and the HR at the **upper right is row-to-column ratio**. Data with statistical significance are marked in bold. NA, not available; T-DM1, trastuzumab emtansine; T-DXd, trastuzumab deruxtecan; RC48, disitamab Vedotin; Chem + TKI, chemotherapy combined with tyrosine kinase inhibitors..

Table S2. Comparison of the incidence of treatment-related adverse event among treatment groups (%).

| Adverse event type | Grade | T-DM-1 ^a^ | T-DXd | SHR-A1811 | ARX788 | Chemo+TKI | A166 | DP303 |
| --- | --- | --- | --- | --- | --- | --- | --- | --- |
| **Hematologic toxicity** | | | | | | | | |
| Neutropenia | Any grade | 9.325 | 42.8 | 89.4 | 15.5 | 27.17 | NA | NA |
| Neutropenia | Grade ≥3 | 2.67 | 19.1 | 54.2 | 2.3 | 5.53 | NA | NA |
| Leukopenia | Any grade | 7.7 | 30 | 78.2 | 17.7 | 25.16 | NA | NA |
| Leukopenia | Grade ≥3 | 0.4 | 6.6 | 20.4 | 0.5 | 1.24 | NA | NA |
| Thrombocytopenia | Any grade | 41.155 | 24.9 | 35.2 | 29.5 | 7.85 | NA | NA |
| Thrombocytopenia | Grade ≥3 | 19.595 | 7 | 10.6 | 2.7 | 0.95 | NA | NA |
| Anemia | Any grade | 14.04 | 30.4 | 75.4 | 11.4 | 33.63 | NA | NA |
| Anemia | Grade ≥3 | 4.04 | 5.8 | 7.7 | 0.9 | 3.25 | NA | NA |
| **Gastrointestinal toxicity** | | | | | | | | |
| Nausea | Any grade | 34.41 | 72.8 | 64.1 | 13.2 | 41.1 | NA | NA |
| Nausea | Grade ≥3 | 0.61 | 6.6 | 1.4 | 0.5 | 1.75 | NA | NA |
| Vomiting | Any grade | 13.26 | 44 | 32.4 | 9.1 | 35.71 | NA | NA |
| Vomiting | Grade ≥3 | 0.71 | 1.6 | 0.7 | 0 | 2.74 | NA | NA |
| Diarrhea | Any grade | 14.555 | 23.7 | 26.1 | 5 | 71.47 | NA | NA |
| Diarrhea | Grade ≥3 | 1.12 | 0.4 | 0.7 | 0.5 | 16.54 | NA | NA |
| Fatigue | Any grade | 33.22 | 44.7 | - | - | 29.71 | NA | NA |
| Fatigue | Grade ≥3 | 1.625 | 5.1 | - | - | 3.48 | NA | NA |
| **Hepatic impairment** | | | | | | | | |
| ALT increased | Any grade | 23.09 | 19.5 | 47.2 | 59.5 | 28.88 | NA | ≥20 |
| ALT increased | Grade ≥3 | 3.83 | 1.6 | 2.1 | 2.3 | 2.41 | NA | NA - |
| AST increased | Any grade | 31.15 | 23.3 | 44.4 | 70.9 | 36.19 | NA | ≥20 |
| AST increased | Grade ≥3 | 4.745 | 0.8 | 0.7 | 1.4 | 1.81 | NA | NA |
| GGT increased | Any grade | NA | NA | 27.5 | NA | NA | NA | NA |
| GGT increased | Grade ≥3 | NA | NA | 0.7 | NA | 9.7 | NA | NA |
| **Specific/notable toxicities** | | | | | | | | |
| Keratopathy | Any grade | NA | NA | NA | 28.2 | NA | NA | ≥20 |
| Keratopathy | Grade ≥3 | NA | NA | NA | 5.9 | NA | ≥5 | NA |
| Blurred vision | Any grade | NA | NA | NA | 35 | NA | NA | ≥20 |
| Blurred vision | Grade ≥3 | NA | NA | NA | 12.3 | NA | ≥5 | NA |
| Dry eye | Any grade | NA | NA | NA | 55 | NA | NA | ≥20 |
| Dry eye | Grade ≥3 | NA | NA | NA | 9.1 | NA | ≥5 | NA |
| Interstitial lung disease (ILD) | Any grade | 1.9 | 10.5 | 2.8 | 32.7 | NA | NA | NA |
| Interstitial lung disease (ILD) | Grade ≥3 | NA | 0.8 | 0.7 | 5.9 | NA | NA | NA |
| Palmar-plantar erythrodysesthesia syndrome | Any grade | 1.43 | NA | NA | 0 | 57.08 | NA | NA |
| Palmar-plantar erythrodysesthesia syndrome | Grade ≥3 | NA | NA | NA | 0 | 15.11 | NA | NA |

Note: Data are derived from the phase III clinical trial reports of the included studies. NA indicates that the adverse event data were not reported. Data for T-DM1 are the average values from the T-DM1 arms in the EMILIA, DESTINY-Breast03, and KL166-III-06 studies. Data for Chem + TKI are the average values from the chemotherapy plus tyrosine kinase inhibitor arms in the EMILIA, HORIZON-Breast01, and E-Breast-02 studies; T-DM1, trastuzumab emtansine; A166, Trastuzumab botidotin ; T-DXd, trastuzumab deruxtecan; RC48, disitamab Vedotin; Chem + TKI, chemotherapy combined with tyrosine kinase inhibitors..

Table S3. PubMed, Embase, and Cochrane Library search strategy

| Database | Search Strategy | Results (n) | Date |
| --- | --- | --- | --- |
| PubMed | (breast[Title/Abstract] OR mammary[Title/Abstract] OR nipple[Title/Abstract] OR areola[Title/Abstract] OR lobules[Title/Abstract]) AND (cancer[Title/Abstract] OR malignancy[Title/Abstract] OR malignant[Title/Abstract] OR tumor[Title/Abstract] OR neoplasm[Title/Abstract] OR carcinoma[Title/Abstract] OR sarcoma[Title/Abstract] OR oncology[Title/Abstract] OR adenocarcinoma[Title/Abstract]) AND (metastasis[Title/Abstract] OR advanced[Title/Abstract] OR metastases[Title/Abstract] OR metastatic[Title/Abstract] OR metastasize[Title/Abstract] OR disseminated[Title/Abstract] OR secondary[Title/Abstract] OR spread[Title/Abstract] OR recurrent[Title/Abstract]) AND (HER-2[Title/Abstract] OR human epidermal growth factor receptor 2[Title/Abstract] OR HER2[Title/Abstract] OR ERBB2[Title/Abstract] OR neu[Title/Abstract]) AND (positive[Title/Abstract] OR overexpressing[Title/Abstract] OR overexpressed[Title/Abstract] OR high[Title/Abstract] OR elevated[Title/Abstract] OR increased[Title/Abstract]) AND (randomized controlled trial[Publication Type] OR RCT[All Fields] OR randomized[All Fields] OR randomised[All Fields] OR random allocation[MeSH Terms]) | 1663 | 31 Dec 2025 |
| Embase | ('breast'/exp OR 'breast':ti,ab OR 'mammary':ti,ab OR 'nipple':ti,ab OR 'areola':ti,ab OR 'lobules':ti,ab) AND ('cancer'/exp OR 'cancer':ti,ab OR 'malignancy':ti,ab OR 'malignant':ti,ab OR 'tumor':ti,ab OR 'neoplasm':ti,ab OR 'carcinoma':ti,ab OR 'sarcoma':ti,ab OR 'oncology':ti,ab OR 'adenocarcinoma':ti,ab) AND ('metastasis'/exp OR 'metastasis':ti,ab OR 'advanced':ti,ab OR 'metastases':ti,ab OR 'metastatic':ti,ab OR 'metastasize':ti,ab OR 'disseminated':ti,ab OR 'secondary':ti,ab OR 'spread':ti,ab OR 'recurrent':ti,ab) AND ('HER-2'/exp OR 'HER-2':ti,ab OR 'human epidermal growth factor receptor 2':ti,ab OR 'HER2':ti,ab OR 'ERBB2':ti,ab OR 'neu':ti,ab) AND ('positive':ti,ab OR 'overexpressing':ti,ab OR 'overexpressed':ti,ab OR 'high':ti,ab OR 'elevated':ti,ab OR 'increased':ti,ab) AND ('randomized controlled trial'/exp OR 'rct':ti,ab OR 'randomized':ti,ab OR 'randomised':ti,ab OR 'random allocation':ti,ab) | 6156 | 31 Dec 2025 |
| Cochrane Library | (breast OR mammary OR nipple OR areola OR lobules) AND (cancer OR malignancy OR malignant OR tumor OR neoplasm OR carcinoma OR sarcoma OR oncology OR adenocarcinoma) AND (metastasis OR advanced OR metastases OR metastatic OR metastasize OR disseminated OR secondary OR spread OR recurrent) AND (HER-2 OR human epidermal growth factor receptor 2 OR HER2 OR ERBB2 OR neu) AND (positive OR overexpressing OR overexpressed OR high OR elevated OR increased) AND (randomized controlled trial OR RCT OR randomized OR randomised OR random allocation) | 4490 | 31 Dec 2025 |
